# MFPNet: A Semantic Segmentation Network for Regular Tunnel Point Clouds Based on Multi-Scale Feature Perception

**DOI:** 10.3390/s26030848

**Published:** 2026-01-28

**Authors:** Junwei Tong, Min Ji, Pengfei Song, Qiang Chen, Chun Chen

**Affiliations:** 1 College of Geodesy and Geomatics, Shandong University of Science and Technology, Qingdao 266590, China; 202382020064@sdust.edu.cn (J.T.); jimin@sdust.edu.cn (M.J.); 2 Qingdao Key Laboratory of Beidou Navigation and Intelligent Spatial Information Technology Application, Qingdao 266590, China; 3 Shandong Engineering Research Center for Beidou Navigation and Intelligent Spatial Information Technology Application, Qingdao 266590, China; 4 Yankuang Energy Group Co., Ltd., Jining No.3 Coal Mine, Jining 272000, China

**Keywords:** tunnel point clouds, semantic segmentation, multi-scale perception, feature fusion

## Abstract

Tunnel point cloud semantic segmentation is a critical step in achieving refined perception and intelligent management of tunnel structures. Addressing common challenges including indistinct boundaries and fine-grained category discrimination, this paper proposes MFPNet, a multi-scale feature perception network specifically designed for tunnel scenarios. This approach employs kernel convolution to effectively model local point cloud geometries within continuous spaces. Building upon this foundation, an error-feedback-based local-global feature fusion mechanism is designed. Through bidirectional information exchange, higher-level semantic information compensates for and constrains lower-level geometric features, thereby mitigating information fragmentation across semantic hierarchies. Furthermore, an adaptive feature re-calibration and cross-scale contextual correlation mechanism is introduced to dynamically modulate multi-scale feature responses. This explicitly models contextual dependencies across scales, enabling collaborative aggregation and discriminative enhancement of multi-scale semantic information. Experimental results on tunnel point cloud datasets demonstrate that the proposed MFPNet has achieved significant improvements in both overall segmentation accuracy and category balance, with mIoU reaching 87.5%, which is 5.1% to 33.0% higher than mainstream methods such as PointNet++ and RandLA-Net, and the overall classification accuracy reaching 96.3%. These results validate the method’s efficacy in achieving high-precision three-dimensional semantic understanding within complex tunnel environments, providing robust technical support for tunnel digital twin and intelligent detection applications.

## 1. Introduction

Underground space constitutes a vital component of urban infrastructure development and resource utilization. Among its diverse forms, tunnels represent one of the most characteristic and significant spatial structures. They are not only extensively applied in engineering domains such as urban rail transit [1], municipal utility tunnels [2] and water conservancy facilities [3], but also play an irreplaceable role in mining resource extraction [4,5,6]. Typically situated at considerable depths underground, tunnels operate within complex environments, feature extensive structural systems, and present significant construction challenges. Once completed, they are expected to sustain decades or even centuries of intensive and frequent operation. Any structural deformation, leakage, settlement, cracking, or equipment ageing can readily trigger operational failures or even safety incidents, resulting in substantial economic losses and societal repercussions [7,8,9]. Precise semantic segmentation of critical tunnel structural components constitutes a key technology underpinning tunnel structural health assessment, deformation monitoring, crack localization, and parametric three-dimensional reconstruction [10].

Traditional tunnel semantic segmentation methods are primarily based on image segmentation techniques. Specifically, images of tunnel structural surfaces are acquired using cameras, and semantic segmentation and recognition are performed either through manual interpretation [11,12,13,14] or computer algorithms [15,16,17,18,19]. Manual processing of image data relies heavily on extensive professional experience and knowledge, which introduces significant subjectivity and is difficult to meet the timeliness requirements of high-frequency tunnel inspections. Although deep learning techniques enable automated processing of image data and can substantially reduce manual effort and time costs, the subway tunnel images collected often fail to capture the complete tunnel structure and are easily affected by uneven illumination conditions, posing dual limitations. In contrast, three-dimensional (3D) laser scanning technology offers rapid acquisition, high fidelity, and rich detail representation, enabling faithful reconstruction of complex tunnel spatial structures. This technique can achieve large-scale, high-density 3D point cloud acquisition of tunnel spaces within a short time, comprehensively recording both geometric forms and subtle features of tunnel structures [20]. Unlike image acquisition methods that rely on external lighting, point cloud acquisition does not require additional light sources [21] and is unaffected by illumination conditions, allowing stable data capture even in completely dark or unevenly lit environments. These characteristics make point cloud data particularly advantageous for high-precision tunnel modeling [22], deformation monitoring [23], and accurate segmentation tasks.

With the continuous development of 3D point cloud acquisition and processing technologies, research on point cloud data has gained increasing prominence in areas such as underground space modeling, structural analysis, and semantic understanding [24,25,26,27,28]. Among these, point cloud semantic segmentation, as a critical component for achieving refined tunnel perception, has emerged as a current research focus. Existing methods for tunnel point cloud semantic segmentation predominantly concentrate on strategies that indirectly convert raw point clouds into images or voxels.These methods that project point clouds onto images or perform voxelization generally suffer from issues such as geometric information loss, boundary blurring, and reduced structural resolution. Consequently, a more effective approach is needed for processing point clouds. Point-based segmentation methods enable direct feature learning on point clouds without requiring additional transformations [29,30], and they have a natural advantage in maintaining the integrity of the original geometric structure.However, due to the existence of various structural types, significant scale differences, and complex spatial relationships in the tunnel environment, the existing methods still struggle to simultaneously achieve both segmentation accuracy and spatial consistency in the tunnel semantic segmentation task. To address these issues, this paper proposes a refined tunnel semantic segmentation network that directly processes unstructured point clouds. Specifically:

(1) A Kernel Point Convolution (KPConv) architecture integrating multi-scale spatial features is designed, which unifies standard KPConv, Strided KPConv, and Multi-Scale KPConv into a bottom-up feature extraction framework combining dense and sparse representations. This enhances semantic modeling capabilities for structurally diverse targets across scales.
(2) A local-global feature fusion module based on error feedback was constructed. By introducing global context information to explicitly compensate and constrain the local features, it effectively alleviated the problems such as blurred boundaries and difficulty in distinguishing small-scale categories in tunnel scenarios, and improved the consistency and discriminability of multi-layer feature representations.
(3) Introduce a dual-branch enhancement mechanism based on feature modulation. By jointly modeling the semantic correlation of the channels and the spatial response distribution, the multi-scale fused features are adaptively re-calibrated and structurally enhanced, thereby further strengthening the discriminative semantic expression of key components.

## 2. Related Work

### 2.1. Projection-Based or Voxel-Based Methods

Ji et al. [31] designed an encoder-decoder [32,33] point cloud semantic segmentation network tailored for metro tunnel environments. This approach first voxelizes raw point clouds and then employs 3D convolutional networks to extract spatial features, enabling multi-class prediction of tunnel objects with robust global structural modelling capabilities. Zhang et al. [34] considering the diversity and complexity of structural components and leakage-related defects in tunnel scenes, proposed an integrated deep learning segmentation framework that combines projection-based two-dimensional (2D) image segmentation methods with direct point cloud processing strategies. By leveraging multimodal feature complementarity, their method improves segmentation accuracy across multiple object categories. The lightweight LCJ-Seg network proposed by Tan et al. [35] similarly employs voxelization for preprocessing and incorporates a local-global cross-attention module. The projection- and voxelization-based point cloud semantic segmentation methods transform unstructured point clouds into regularized representations, which facilitates employing mature 2D/3D convolutional networks for modeling global spatial structures. However, the regular grid reconstruction process often leads to loss of geometric details and multi-scale information, consequently limiting fine-grained segmentation performance for small objects and semantic boundaries in complex tunnel environments.

### 2.2. Point-Based Methods

Qi et al. [36] pioneered PointNet, which for the first time achieved end-to-end direct learning on raw point clouds. By incorporating a symmetric function mechanism, it accommodates the inherent disorder of input data, thereby overcoming traditional convolutional neural networks’ reliance on regular grid structures. Nevertheless, this method lacks the ability to effectively model local geometric structures and struggles to capture fine-grained spatial features. PointNet++ [37] introduced hierarchical sampling and local feature extraction mechanisms to enhance local geometric modeling capabilities. However, it incurs high computational overhead when processing large-scale point clouds and exhibits performance sensitivity under sparse data conditions. RandLA-Net proposed by Hu et al. [38] combines local feature aggregation with an attention mechanism, significantly improving semantic representation capabilities while maintaining computational efficiency. It is particularly suited for processing large-scale sparse point clouds, though its ability to preserve feature details remains limited when dealing with complex topological structures or small-scale objects. Wang et al. [39] proposed DGCNN, which captures local topological variations through dynamic graph construction and EdgeConv operations, demonstrating excellent performance in point cloud semantic segmentation tasks. While demonstrating outstanding performance in point cloud semantic segmentation tasks, its dynamic graph updating process incurs high computational and memory costs, resulting in efficiency bottlenecks in ultra-large-scale point cloud scenarios. Although the aforementioned point-based methods have demonstrated outstanding performance in fields such as indoor scene analysis [40,41], autonomous driving [42,43,44], and large-scale outdoor point cloud segmentation [45], effectively mitigating the widespread issues of geometric information loss and feature deficiency inherent in projection-based and voxelization approaches, their performance often fails to maintain comparable levels in underground environments such as tunnels, which are structurally enclosed, morphologically complex, and characterized by highly imbalanced category distributions. In such scenarios, these methods frequently encounter problems including blurred segmentation boundaries, missed detections of small objects, and insufficient overall accuracy, with significant shortcomings in small-scale object recognition, boundary prediction, and maintaining long-range structural continuity.

## 3. Materials and Methods

### 3.1. Dataset

**Figure 1:**
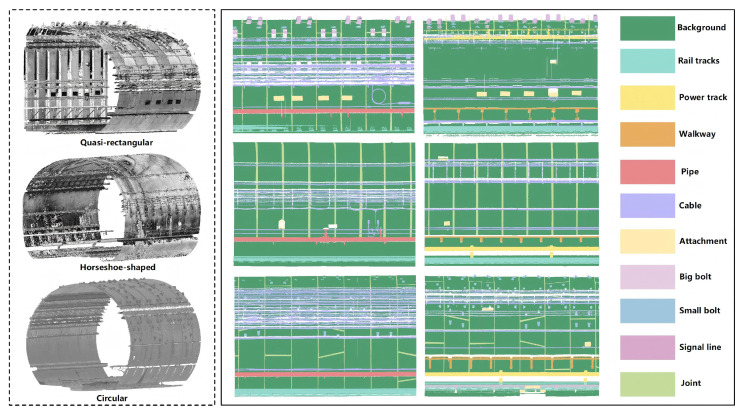
Annotations of the STSD dataset.

**Figure 2:**
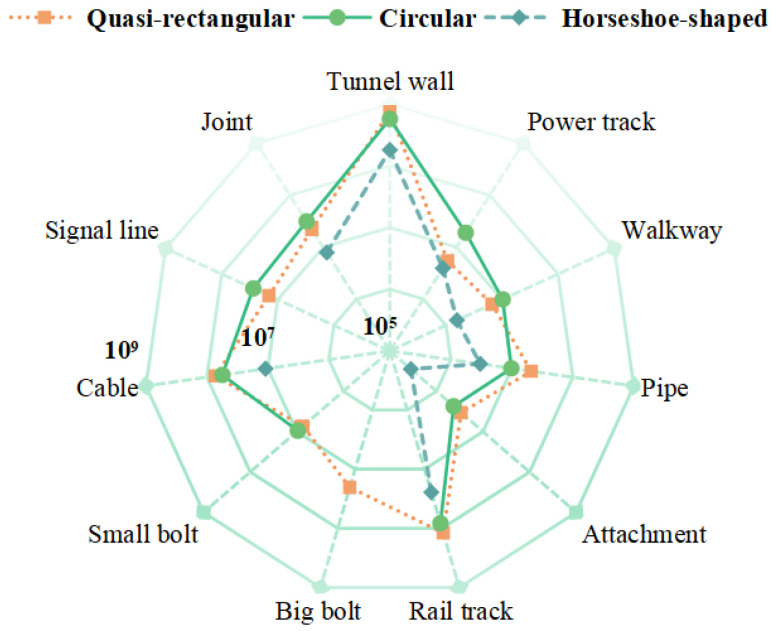
Label distribution of the STSD dataset.

This study employs regular tunnel data as an example, with experimental data sourced from the large-scale subway tunnel semantic segmentation dataset (STSD) proposed by Zhengzhou University in China [46]. Constructed from point cloud data of Wuhan Metro tunnels acquired via Mobile Laser Scanning (MLS) systems, this dataset encompasses three typical cross-sectional tunnel types: horseshoe-shaped, quasi-rectangular, and circular. The total scanned length reaches 2700 m, comprising approximately 2.26 billion 3D point cloud points. Moreover, the STSD dataset consists of 11 categories: Background, Rail tracks, Power track, Walkway, Pipe, Cable, Big bolt, Small bolt, Signal line, Joint, and Attachment. The latter denotes ancillary equipment affixed to tunnel walls. The specific annotation distribution across categories is illustrated in Figure 1. In tunnel scenes, tunnel wall categories constitute the overwhelming majority, while categories such as Walkway and Power track account for a relatively small proportion. Figure 2 illustrates the distribution of each category across the three tunnel types, revealing an issue of data sample imbalance across categories within tunnel scenarios.

### 3.2. MFPNet Network Architecture

**Figure 3:**
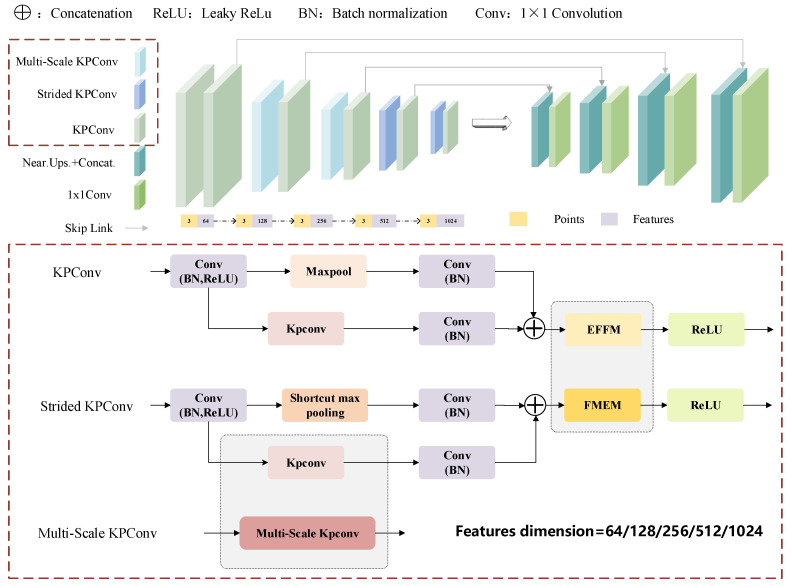
Overview of the proposed MFPNet.

This study proposes a refined tunnel semantic segmentation network that operates directly on unstructured point clouds. The specific MFPNet workflow is illustrated in Figure 3. The network architecture incorporates the synergistic application of multiple kernel point convolution (KPConv) structures, including standard KPConv, Strided KPConv, and Multi-Scale KPConv, each undertaking feature extraction tasks at distinct hierarchical levels. Standard KPConv preserves local geometric details, proving suitable for capturing fine-grained semantic information in dense regions. Strided KPConv effectively expands the receptive field through downsampling operations, extracting structural features from sparse areas. Multi-Scale KPConv further integrates outputs from multiple scale branches, enhancing the network’s perception of targets across varying spatial scales. Subsequently, an Error Feedback Fusion Module (EFFM) is constructed at a specific encoding layer. By explicitly modeling the implicit relationship between local features and global semantics, this module employs an error feedback mechanism to propagate high-level global attention information back to the original local features, thereby significantly enhancing local semantic representation. During the feature fusion stage, a Feature Modulation and Enhancement Module (FMEM) is incorporated. This module jointly employs channel attention and spatial attention mechanisms to mine multi-scale contextual relationships, enabling precise localization and classification of small objects within complex tunnel environments. Through the synergistic action of multi-scale feature extraction, error feedback enhancement, and dual attention enhancement, the entire network substantially improves segmentation performance for fine-grained semantics while preserving structural integrity.

Moreover, as underground railway tunnels are typically situated at considerable depths beneath the surface, their internal illumination conditions are extremely limited. This results in data acquired via laser scanning systems often exhibiting significant gaps or substandard quality within the RGB color channels [47]. Therefore, to avoid interference introduced by inaccurate or unstable RGB features, and considering that compressing feature dimensions reduces computational complexity in subsequent processing, this study omits RGB information in feature design. Instead, point cloud intensity values are incorporated, enabling each point cloud to comprise spatial coordinates and intensity features. This approach enhances the network’s robustness and efficiency.

#### 3.2.1. Multi-Scale Kernel Point Convolution

Kernel Point Convolution is a spatial convolution operation applied to point cloud data. Unlike traditional grid-based convolutions (Conv), it does not rely on voxelization or projection. Instead, it performs local feature extraction directly on irregular point clouds in three-dimensional space through learnable kernel points [48].

KPConv can be generally formulated as a point convolution operation, as shown in Equation (1). Typically, the points on the input data $P=\left\{x_{i}|i=1,2,\ldots ,N\right\}\in R^{N\times 3}$ and their corresponding features in $F=\left\{f_{i}|i=1,2,\ldots ,N\right\}\in R^{N\times D}$ are denoted as $x_{i}$ and $f_{i}$, where N represents the number of points and $x_{i}$ denotes the $\left(x,y,z\right)$ coordinates of the *i*-th point.The general point convolution of F at point $x\in R^{3}$ for the input point cloud is defined as:

(1) $$F\left(x\right)=\sum_{x_{i}\in N_{x}}\sum_{k=1}^{K}h\left(x_{i}-x,{\tilde{x}}_{k}\right)W_{k}f_{i}$$

Here, $W_{k}$ denotes the weight matrix mapping the feature vector dimensions from $D_{in}$ to $D_{out}$, while *h* represents the proportional coefficient between the kernel point ${\tilde{x}}_{k}$ and the three-dimensional coordinate point $y_{i}$ in the local coordinate system. When the latter two exhibit spatially similar shapes, drawing inspiration from bilinear interpolation, the linear coefficient *h* can be expressed by the following equation:

(2) $$h\left(x_{i}-x,{\tilde{x}}_{k}\right)=max\left(0,1-\frac{∥y_{i}-{\tilde{x}}_{k}∥}{\sigma }\right)$$

where σ is the influence radius of a kernel point, and will be determined according to the density of the input points.

However, the traditional single-scale kernel point convolution is constrained by its fixed-radius neighborhood, making it difficult to simultaneously model features of both large-scale structures (e.g., tunnel walls) and small target components (e.g., pipes and cables) within tunnel point cloud data. This results in reduced segmentation accuracy in complex scenarios. Although the original authors introduced the deformable kernel point convolution, which adaptively adjusts the convolution region by learning spatial offsets of kernel points to enhance the network’s perception of non-rigid structures and boundary areas, it still suffers from numerous limitations. Specifically, the kernel points lack explicit geometric constraints, the training process is unstable, and the scale modeling capacity remains insufficient, making it challenging to systematically capture multi-granularity structural information. To address these issues, this paper proposes a multi-scale kernel point convolution architecture. By employing multi-radii kernel point convolution, it enhances modeling capabilities for structures with varying receptive fields, thereby improving the network’s semantic segmentation accuracy in complex underground environments.

**Figure 4:**
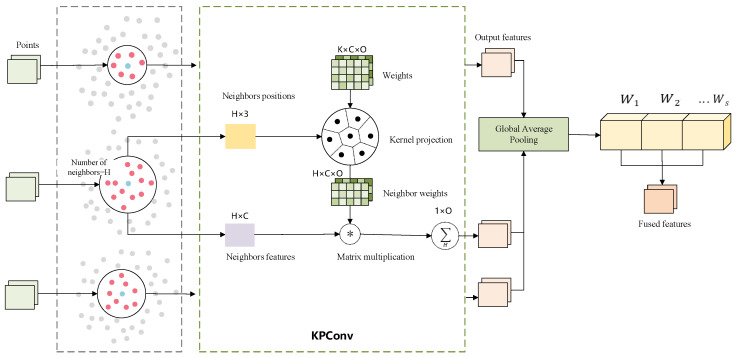
Architectural diagram of the Multi-Scale KPConv Module.

Multi-scale KPConv extracts local features at different scales through multiple receptive fields, enhancing the representation of structural details and global context. As illustrated in Figure 4, for *S* distinct scales $r_{1},r_{2},\ldots ,r_{s}$, KPConv is executed once per scale, with each scale possessing an independent set of kernel points ${\tilde{x}}_{k}^{\left(s\right)}$ and weight matrices ${W_{k}}^{\left(s\right)}$:

(3) $$F^{\left(S\right)}\left(x\right)=\sum_{x_{i}\in {N_{x}}^{\left(S\right)}}\sum_{k=1}^{K_{\left(S\right)}}h^{\left(S\right)}\left(x_{i}-x,{\tilde{x}}_{k}^{\left(s\right)}\right){W_{k}}^{\left(S\right)}f_{i}$$

To fully exploit the features extracted at different scales, this paper introduces a channel attention mechanism for weighted fusion of multi-scale features. First, feature values from all points at each scale undergo average pooling along the channel dimension. Then, the averaged results across all scales are aggregated to obtain the channel statistical description vector $\bar{z}$, defined as shown in Equation (4):

(4) $$\bar{z}=\frac{1}{S}\sum_{s=1}^{S}\left(\frac{1}{N}\sum_{i=1}^{N}F_{i}^{\left(s\right)}\right)$$

Subsequently, the channel attention module adaptively learns the importance of different scales based on this descriptor, yielding the weighting coefficients $\alpha \in R^{S}$. The specific calculation process is shown in Equation (5):

(5) $$\alpha =Softmax\left(W_{2}\cdot \sigma \left(W_{1}\cdot \bar{z}\right)\right)$$

Finally, the output features of each scale are aggregated by weighted summation, thereby achieving multi-scale feature fusion, as formulated in Equation (6):

(6) $$F_{\mathrm{fused}}\left(x\right)=\sum_{s=1}^{S}\alpha_{s}\cdot F^{\left(S\right)}\left(x\right)$$

The Multi-Scale KPConv module introduced in MFPNet is designed to comprehensively model the geometric and structural characteristics of tunnel point clouds across different spatial scales. Specifically, at each selected network layer, the Multi-Scale KPConv adopts S = 3 parallel scale branches, each corresponding to a distinct spatial receptive field, in order to capture multi-level feature representations ranging from local geometric details to mid-scale structural semantics. For each scale branch, the neighborhood radius is determined according to the sampling resolution and spatial density of the point cloud at that layer, following the design principle of the original KPConv architecture, in which the convolution radius progressively increases with the downsampling scale. In this setting, smaller-radius neighborhoods are primarily used to capture fine-grained local geometric details, medium-scale neighborhoods are employed to model structural relationships between adjacent components, and larger-radius neighborhoods facilitate the extraction of more stable structural semantic information. The selection of these parameters is intended to ensure geometric consistency between neighborhood coverage and the physical scale of the point cloud at different resolutions, rather than treating the neighborhood radius as an independent hyperparameter for performance tuning. During multi-scale feature extraction, all scale branches share the same set of input points and sampling results, and differ only in the neighborhood query stage by employing different search radii for feature aggregation. This design avoids spatial bias introduced by repeated resampling, while ensuring spatial alignment of features across scales, thereby providing a reliable foundation for subsequent multi-scale feature fusion. It should be noted that the ablation experiments in this study primarily focus on the number of kernel points (K), which has a more direct impact on the expressive capacity of the convolutional kernel. In contrast, the multi-scale neighborhood radii and neighborhood sizes are treated as structure-driven configurations bound to point cloud density and sampling resolution, and are therefore not subjected to systematic ablation analysis in this work.

#### 3.2.2. Error Feedback Fusion Module

Local feature learning based on convolution operators is one of the core approaches in point cloud analysis. However, it still exhibits certain limitations when processing complex geometric structures or high-dimensional data [49]. This paper introduces a local-global feature learning module based on error feature back-projection. This module is designed to explicitly model the semantic discrepancy between high-level global semantic features and low-level local geometric features, and to utilize this discrepancy as a feedback signal for the correction and enhancement of local feature representations. It should be emphasized that the term “error” in this context does not refer to an optimization error at the loss-function level, but rather characterizes the inconsistency between feature representations at different semantic hierarchies. Unlike conventional residual connections or attention-guided feature refinement methods, which primarily reorganize information within the same feature level, the proposed EFFM introduces a top-down semantic feedback mechanism, in which high-level semantic information is explicitly projected back to modulate shallow local representations. This design effectively alleviates the representational mismatch between deeply abstract semantic features and fine-grained local geometric details. In addition, EFFM adopts a lightweight feature transformation and fusion strategy, which significantly enhances feature representation capability while introducing only a minimal number of additional parameters and computational overhead, thereby ensuring the engineering feasibility of the overall network.

**Figure 5:**
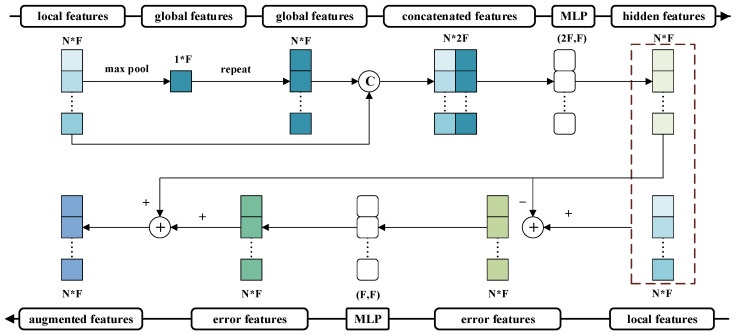
Architectural diagram of the Error Feedback Fusion Module.

As illustrated in Figure 5, the module takes the local features obtained by convolution as input. These local features $f_{i}$ are first aggregated into global features g via the max pooling function. Subsequently, both local and global features are combined and fed into a multi-layer perceptron (MLP) to learn the hidden feature representation $\bar{f_{i}}$, as expressed by the following formula:

(7) $$g=\left(f_{1},f_{2},\ldots ,f_{N}\right)\in R^{1\times F}$$

(8) $$\bar{f_{i}}=MLP\left(f_{i}⊕repeat\left(g\right)\right)\in R^{N\times F}$$

Finally, another MLP is applied to encode the error features computed from both local features and hidden features. The encoded error features are then back-projected to update the original local features, resulting in enhanced local representations $\tilde{f_{i}}$, as formulated in Equation (9):

(9) $$\tilde{f_{i}}=f_{i}+MLP\left(\bar{f_{i}}-f_{i}\right)\in R^{N\times F}$$

In summary, the EFFM module adopts a semantically guided feature reorganization strategy in its architecture, which enables high-level semantic features not only to transmit macro-level semantic information to lower layers but also to receive spatial feedback from lower-level features. In this way, dynamic correction and detail compensation are achieved during multi-scale semantic fusion. This mechanism effectively mitigates inconsistencies between deep abstract semantics and shallow spatial representations, significantly enhancing the network’s ability to analyse tunnel structural details, ambiguous boundary regions, and small targets.

#### 3.2.3. Feature Modulation and Enhancement Module

**Figure 6:**
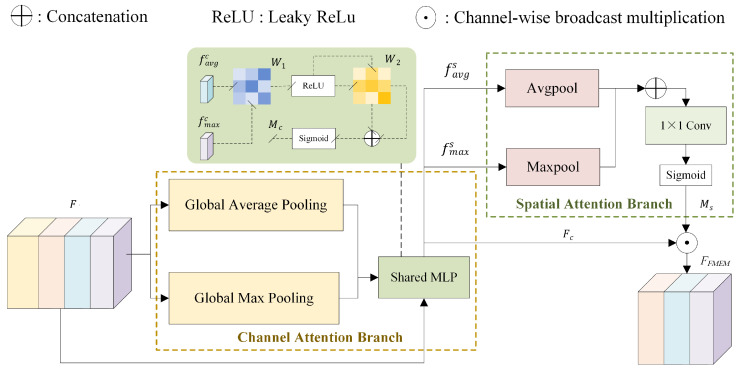
Architectural diagram of the Feature Modulation and Enhancement Module.

Feature representational capability is crucial in point cloud analysis, especially for complex environments such as tunnels. The attention module can dynamically allocate resources during feature representation, enabling the network to focus on more important feature regions or channels, thereby enhancing semantic modeling capability and contextual understanding. To address the issues of class imbalance and insufficient local geometric information in tunnel point clouds, a Feature Modulation and Enhancement Module (FMEM) is proposed in this study, as illustrated in Figure 6. The module explicitly models and adaptively fuses point cloud features from two complementary perspectives, namely the channel dimension and the spatial dimension.

This module does not introduce additional spatial position encoding; instead, it operates directly on the local geometric features extracted by KPConv, and performs adaptive enhancement of feature responses through joint modulation along the channel dimension and the point-wise spatial dimension. Unlike generic attention modules that primarily focus on global feature reweighting, FMEM is designed to further strengthen the discriminative capability of mid- to high-level representations after multi-scale feature fusion, with particular emphasis on geometrically sensitive regions and easily confused structural components. Benefiting from its lightweight and modular design, FMEM can be flexibly embedded after multiple residual blocks within the KPConv encoder. During experimental evaluation, it is observed that introducing FMEM at the 9th and 10th layers leads to a significant improvement in the detection performance of crack-related classes and small target categories. By dynamically adjusting both channel-wise and spatial attention regions in the feature maps, FMEM effectively alleviates insufficient feature representation caused by uneven point cloud density and class imbalance. Notably, these performance gains are achieved without introducing additional parameters or complex architectural components, resulting in a substantial improvement in segmentation performance and robustness, particularly in structurally complex tunnel point cloud scenes with pronounced scale variations. Different channels within a neural network typically correspond to distinct types of semantic features, such as edges, textures, or object shapes. In tunnel point clouds, certain channels may encode features of rails, pipes, or joint structures. Therefore, the channel attention mechanism evaluates “which channels are more important,” reinforcing the responses of key feature channels while suppressing irrelevant or redundant ones. For the input feature $F\in R^{C\times N}$ (where *C* is the number of channels and N is the number of points), average pooling and max pooling are applied along the point dimension to obtain two global descriptors:

(10) $$f_{avg}^{c}=\frac{1}{N}\sum_{i=1}^{N}F_{:,i},f_{max}^{c}=\max_{i=1,\ldots ,N}F_{:,i}$$

These descriptors are then passed through a shared MLP for feature fusion, formulated as:

(11) $$M_{c}=\sigma \left(W_{2}\cdot \delta \left(W_{1}\cdot f_{avg}^{c}\right)+W_{2}\cdot \delta \left(W_{1}\cdot f_{max}^{c}\right)\right)$$

where $W_{1}\in R^{\frac{C}{r}\times C}$ and $W_{2}\in R^{C\times \frac{C}{r}}$ are learnable weight matrices, $\delta \left(\cdot \right)\;$ denotes the ReLU activation function, and $\;\sigma \left(\cdot \right)$ is the Sigmoid function. The channel attention coefficient $M_{c}\in R^{C}$ is multiplied element-wise with the input feature to obtain $F_{C}=M_{C}⊙F$, thereby achieving explicit enhancement of the key feature channels.

The purpose of the spatial attention mechanism is to enable the network to “know where to focus more.” After obtaining the channel-enhanced features $F_{C}$, the spatial attention branch aggregates the channel responses at each point to highlight the spatial saliency distribution. Specifically, for each point, average pooling and max pooling are applied along the channel dimension:

(12) $$f_{avg}^{s}=\frac{1}{C}\sum_{j=1}^{C}F_{j,:},f_{max}^{s}=\max_{j=1,\ldots ,C}F_{j,:}$$

The two results are concatenated along the channel dimension and fed into a 1D convolutional network to generate the spatial attention map:

(13) $$M_{s}=\sigma \left(Conv1D\left(\left[f_{avg}^{s};f_{max}^{s}\right]\right)\right),M_{s}\in R^{N}$$

The final output of the spatially enhanced features is expressed as $F_{FMEM}=F_{c}⊙M_{s}$.

## 4. Experiments and Evaluation

### 4.1. Environment and Data

All experiments in this study were conducted on a workstation equipped with the following specifications: Intel i9-10980XE CPU (64 GB RAM), NVIDIA GeForce RTX 3080Ti GPU (CUDA version), Windows 10 operating system, and Python 3.9.10 programming environment. The network parameters were set as follows: learning rate of 1 × $10^{-3}$, batch size of 4, and 300 training iterations.

**Figure 7:**
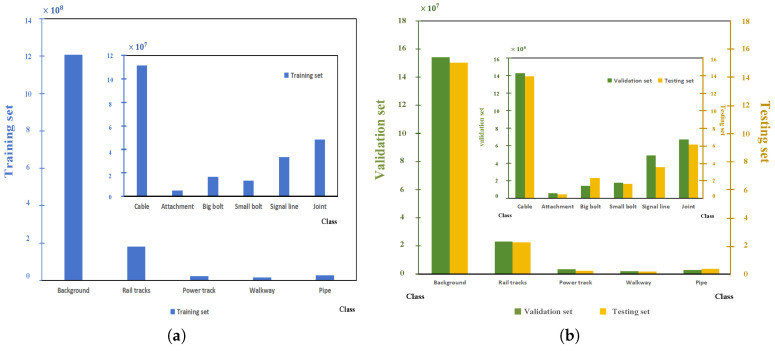
Distribution of different classes in different data set: (**a**) Training and validation sets, (**b**) Testing set.

The dataset used in this study consists of 264 representative metro tunnel point cloud segments, each with a length of approximately 10 m. To ensure the fairness and reproducibility of network training and evaluation, a fixed random seed (seed = 42) was adopted for dataset splitting. The dataset was randomly divided into 211 training samples, 26 validation samples, and 27 test samples, following an 8:1:1 ratio, where the validation and test sets are of comparable size to facilitate a more reliable assessment of model performance in practical application scenarios, as illustrated in Figure 7. It should be noted that the fixed random seed was used solely to determine the dataset partition and to ensure the reproducibility of the experimental setup. Under identical experimental configurations, the data split remains unique and fixed, without introducing additional sources of uncertainty. The dataset was partitioned at the level of tunnel segments, strictly preventing any overlap of data from the same tunnel segment across different subsets, thereby ensuring the independence of the training, validation, and test sets. This partitioning strategy not only preserves scene diversity and structural complexity, but also takes into account the overall balance of semantic class distributions. Consequently, it provides a solid data foundation for training deep learning networks and for evaluating their generalization performance in complex tunnel environments. All experimental results reported in this paper are evaluated based on the fixed data split and unified experimental settings. The performance metrics of different models on the same test set are therefore deterministic, and the reported performance improvements represent absolute performance differences under identical conditions, rather than statistical estimates derived from multiple independent experimental runs.

### 4.2. Evaluation Criteria

To comprehensively evaluate the performance of the MFPNet network across various scenarios and provide a basis for further network optimisation, this study employs the following evaluation metrics: accuracy, precision, recall, Intersection over Union (IoU), and F1score. The formulas for these metrics are as follows:

(14) $$Accuracy=\frac{TP+TN}{TP+TN+FP+FN}$$

(15) $$Precision=\frac{TP}{TP+FP}$$

(16) $$Recall=\frac{TP}{TP+FN}$$

(17) $$IoU=\frac{TP}{TP+FN+FP}$$

(18) $$F1\;score=\frac{2\times TP}{2\times TP+FN+FP}$$

where TP, TN, FP, and FN denote true positives (the number of samples that are genuinely positive and correctly predicted as positive), true negatives (the number of samples that are genuinely negative and correctly predicted as negative), false positives (the number of samples that are genuinely negative but incorrectly predicted as positive), and false negatives (the number of samples that are genuinely positive but incorrectly predicted as negative).

### 4.3. Results and Analysis

**Figure 8:**
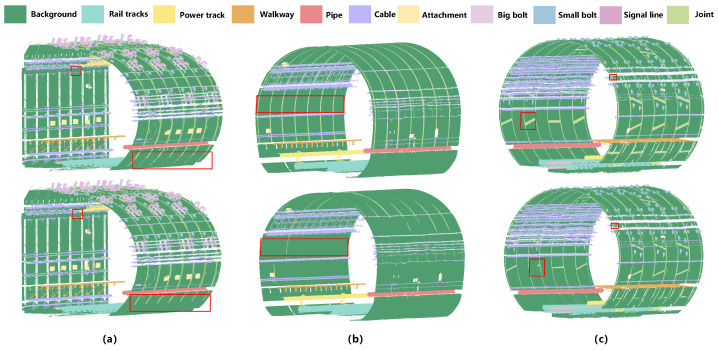
Comparison of semantic segmentation results of MFPNet on different tunnel structural types: (**a**) Quasi-rectangular tunnel, (**b**) Horseshoe-shaped tunnel, and (**c**) Circular tunnel. For each group of images, the results are arranged from top to bottom as ground truth and predicted results, respectively.

Figure 8 presents the visualization results of the proposed network on point cloud semantic segmentation tasks across different types of underground tunnels. It can be observed that MFPNet achieves favorable segmentation performance in typical tunnel structures such as quasi-rectangular, horseshoe-shaped, and circular tunnels. The network is able to accurately distinguish the major tunnel components, including tracks, pipes, and cables, with predictions largely consistent with the ground truth, demonstrating strong structural recognition and semantic representation capabilities. Notably, in circular and quasi-rectangular tunnels, MFPNet exhibits high stability, particularly excelling in boundary sharpness and the restoration of small objects (e.g., bolts and signal lines). Even in challenging regions with weak structural continuity and blurred boundaries, such as tunnel joints, the network can effectively recover semantic boundaries, showcasing strong geometric adaptability and fine-detail perception. However, in horseshoe-shaped tunnels, the segmentation accuracy around tunnel joints is relatively lower, with instances of missed segmentation. This represents one of the primary reasons for the overall lower segmentation accuracy within this category. Furthermore, despite its generally sound performance, MFPNet still exhibited slight under-segmentation for certain small categories. This indicates that the network retains scope for further improvements when handling extremely small objects or regions with blurred semantic boundaries and minimal inter-class distinctions.

**Figure 9:**
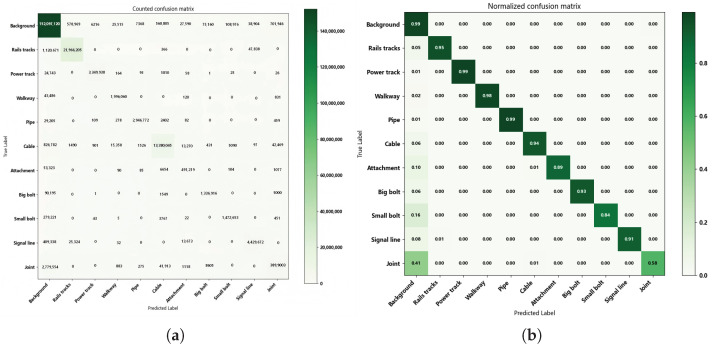
Confusion matrix of the testing point cloud: (**a**) Counted confusion matrix, (**b**) Normalized confusion matrix.

To further demonstrate the segmentation performance of the MFPNet architecture, Figure 9 presents the confusion matrices (including raw and normalized matrices) to intuitively illustrate the misclassification patterns of each category. The results show that most major structural classes achieve high recognition accuracy, with diagonal elements in the normalized confusion matrix generally exceeding 0.95. This indicates the network possesses robust recognition capability and stability for regular geometric shapes, contiguous regions, and large-scale objects. For medium-sized structural targets such as Cable, Attachment, Big bolt, and Signal line, the recognition accuracy also reaches a high level, suggesting that the network possesses strong local semantic perception capability in regions with significant geometric variation or elongated structures. However, for small-volume categories such as Small bolt and Joint, which are often characterized by fuzzy boundaries or occlusion, the performance is relatively weaker. The classification accuracy of Small bolt drops considerably, while the recognition accuracy of Joint is only 0.61, with a large proportion of its instances incorrectly classified as Background in the raw confusion matrix. Furthermore, the non-diagonal regions reveal considerable confusion between certain minor categories. For instance, Small bolt is frequently confused with Background, while Joint exhibits substantial cross-category bias with multiple classes. This indicates that the network still possesses room for improvement in small object detection, boundary discrimination, and fine-grained category differentiation.

Table 1 further corroborates the observations from the confusion matrices by reporting quantitative evaluation metrics, including accuracy, recall, F1-score, and IoU, on the test set. It is evident that the MFPNet achieves relatively favourable segmentation performance across most categories, with metrics exceeding 90% for several key structural classes. Notably, the IoU for regular structures such as Power tracks, Pipes, and Walkways surpasses 95%, fully demonstrating the network’s stability and robustness in primary structural recognition tasks.

### 4.4. Comparative Experiment

To validate the effectiveness of the proposed network, this study conducted systematic comparative experiments against multiple mainstream methods on the STSD dataset. To ensure fairness in the comparative experiments, all evaluated networks uniformly adopted identical data preprocessing procedures and training strategies, with network training and testing performed under identical training iterations and hyperparameter settings.

As shown in Table 2, the mean Intersection over Union (mIoU) results reveal significant differences in overall performance and fine-grained categories among the methods. MFPNet achieved the best performance, reaching an overall mIoU of 87.5%, substantially surpassing other approaches. Specifically, KPConv [48], benefiting from kernel point convolution, effectively modeled local geometric features and achieved nearly 98% IoU in large categories such as Background, Pipe, and Walkway. However, it exhibits limitations in small-object and boundary-sensitive categories like Small bolt and Joint. Point Transformer demonstrates strong global modeling capabilities, achieving an overall mIoU of 77.8% and high segmentation accuracy in elongated categories such as Rail tracks and Cable. Among lightweight methods, SCF-Net [50] and RandLA-Net [38] achieved mIoUs of 75.6% and 73.9%, respectively, with similar performance on large categories. SCF-Net was more robust in handling small-object and boundary categories, while RandLA-Net was relatively weaker in these cases. BAAF-Net [51]’s overall performance is comparable to RandLA-Net, with improvements on boundary categories like cables and signal lines, though it was slightly inferior to SCF-Net on large-scale categories such as walkways. In contrast, the traditional baselines PointNet++ [37] and DGCNN [39] delivered limited results, with mIoUs of 54.5% and 61.8%, respectively. PointNet++ performed reasonably on large categories but failed almost entirely on small ones, while DGCNN showed slight improvements in boundary categories but overall gains were limited.

**Figure 10:**
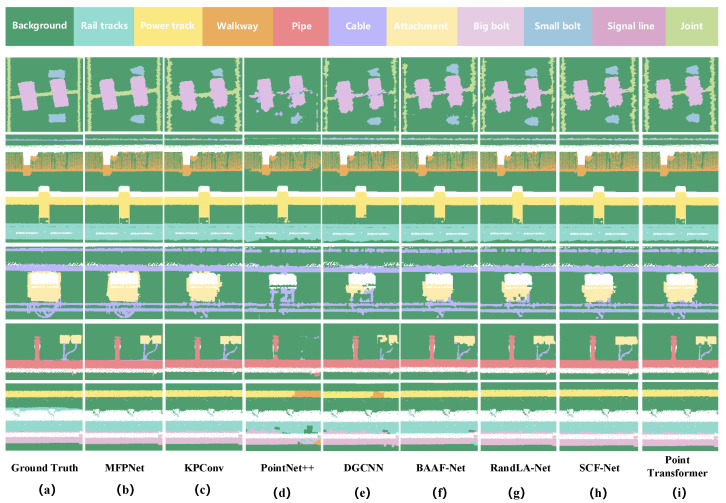
Semantic segmentation results of point clouds across different networks: (**a**) Ground truth; (**b**) MFPNet; (**c**) KPConv; (**d**) PointNet++; (**e**) DGCNN; (**f**) BAAF-Net; (**g**) RandLA-Net; (**h**) SCF-Net; (**i**) Point Transformer.

The qualitative results in Figure 10 further corroborate these conclusions: PointNet++ and DGCNN often suffer from missing small categories and blurred boundaries. RandLA-Net and SCF-Net produce relatively complete segmentation for large categories but failed to recognize small targets such as Big bolt and Signal line. KPConv shows strong performance on local structures such as Bolt and Pipe, yet struggles with the extremely slender Joint. Point Transformer [52] maintains good overall consistency but exhibits discontinuities and noise in small object predictions. In contrast, MFPNet consistently produced stable segmentation for both large-scale categories and small objects, accurately reconstructing key structures such as Pipe, Signal line, and Joint with clear boundaries and complete details. In summary, experimental results demonstrate that MFPNet achieves dual improvements in both accuracy and robustness for tunnel point cloud semantic segmentation. In particular, it exhibits significant advantages in small-object recognition and boundary-sensitive categories, thereby validating the effectiveness and applicability of the proposed method.

### 4.5. Ablation Experiment

**Figure 11:**
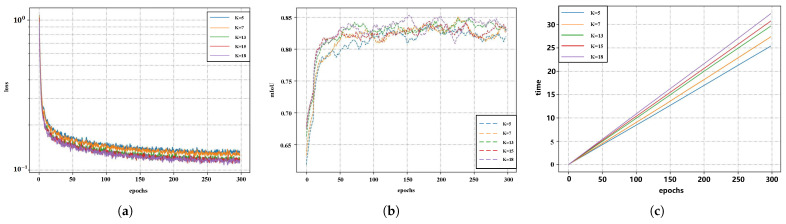
Experimental results with different K values: (**a**) Training loss curves; (**b**) Validation mIoU curves; (**c**) Training time comparison.

To determine the appropriate number of kernel points, multiple experiments were conducted with different values of K, and the results were systematically analyzed in terms of segmentation accuracy, training time, and loss convergence characteristics. Results from the training phase in Figure 11 indicate that the network achieves stable convergence after approximately 50 epochs across all K values. With the increase of K, the overall mIoU exhibited an upward trend. In particular, the mIoU curves for K = 13, K = 15, and K = 18 were consistently higher and less volatile than those for K = 5 and K = 7, demonstrating better stability. Among them, K = 18 achieved the highest peak values at several stages, indicating that larger neighborhoods help capture richer contextual information and thus enhance segmentation performance. However, the difference in final stable accuracy between K = 13 and K = 18 was less than 0.5%, while the training time increased by approximately 18–20%. In terms of loss convergence, all groups showed a rapid decrease in the early stage of training and reached a steady state in the mid-to-late stages, suggesting that varying the number of kernel points does not affect the convergence behavior of the network. Taking both accuracy improvement and computational cost into consideration, although K = 18 yielded marginally higher accuracy at certain stages, K = 13 achieved nearly the same accuracy while significantly reducing training and inference time. This makes it more suitable for deployment in practical scenarios where computational resources are limited or a balance between efficiency and performance is required. Consequently, K = 13 was selected as the final kernel point setting in subsequent experiments to achieve an optimal trade-off between performance and efficiency.

**Figure 12:**
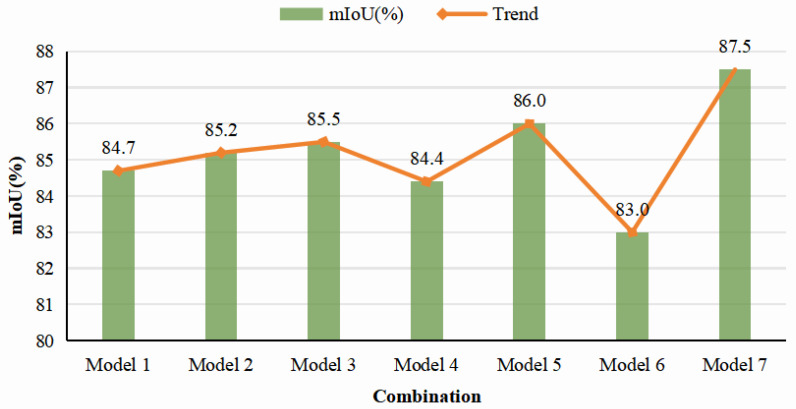
Results of different module combinations.

To systematically evaluate the impact of each functional module within the MFPNet architecture on segmentation performance, this study designs seven sets of ablation experiments with different module combinations, and the results are shown in Figure 12 and Table 3. Overall, each module contributes to varying degrees of improvement to the network’s segmentation accuracy. Incorporating Multi-Scale KPConv alone (Model 1) achieves an mIoU of 84.7% on the baseline architecture, demonstrating its significant contribution in capturing multi-level spatial features. When introducing either EFFM (Model 2) or FMEM (Model 6) individually, the mIoU reaches 85.2% and 83.0% respectively, validating their effectiveness in feature fusion and fine-grained structural modeling. Model 3, which combines Multi-Scale KPConv with EFFM, further increases the mIoU to 85.5%, while the combination of Multi-Scale KPConv and FMEM (Model 5) performs even better, reaching 86.0%. When all three modules were integrated (Model 7), the network achieves the highest mIoU of 87.5%, representing an improvement of 1.5–4.5% compared with networks using individual modules. These results clearly demonstrate the complementary strengths and synergistic effects of the three modules in feature extraction, information fusion, and local detail enhancement.

## 5. Discussion

### 5.1. Joint Omission in Horseshoe-Shaped Tunnel Structures

**Figure 13:**
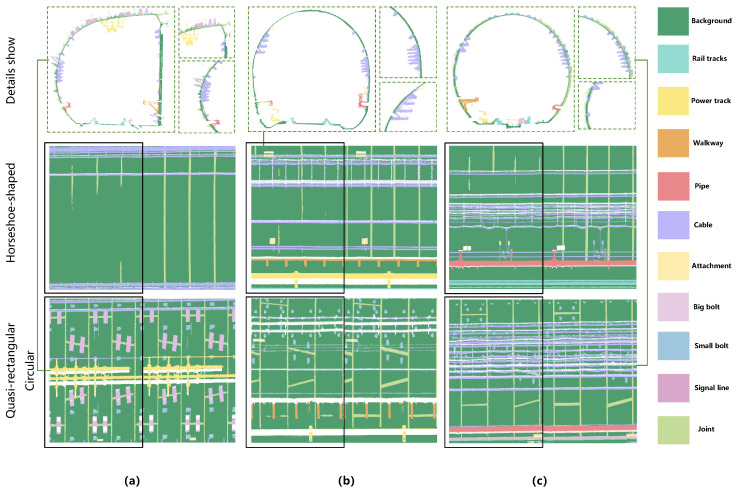
Comparison of joint segmentation results under multiple views for different tunnel structural types. (**a**) Upward view; (**b**) Left view; (**c**) Right view. For each group of images, the top-to-bottom order corresponds to horseshoe-shaped, quasi-rectangular, and circular tunnels. The results inside the black boxes represent the network predictions, while those outside the boxes are the ground truth.

Across tunnels with different cross-sectional shapes, the segmentation of joints in horseshoe-shaped tunnels performs noticeably worse than in quasi-rectangular and circular tunnels, often exhibiting omissions or misclassifications. This is the main reason for the overall lower accuracy of joint segmentation. As illustrated in Figure 13, given the high proportion of pipeline structures in the side view of quasi-rectangular tunnels, circular and horseshoe-shaped tunnels were selected for analysis in the left-right view comparison. Conversely, for the top-view comparison, quasi-rectangular and horseshoe-shaped tunnels were chosen since the top-view morphology of quasi-rectangular tunnels is more representative.

The diminished joint segmentation performance in horseshoe cross-sections stems primarily from the following factors: Firstly, as shown in the detailed view of Figure 13, the transition between the joint and the tunnel wall in horseshoe cross-sections is smoother, with minimal variation in local normal vectors and curvature, making it difficult to extract distinctive geometric cues for boundary discrimination. Secondly, these cross-sections often lack auxiliary structures such as bolts and attachments in the joint area, preventing the network from leveraging contextual information for spatial localization. By contrast, quasi-rectangular and circular tunnels usually exhibit pronounced geometric discontinuities and abundant auxiliary structures around the joint regions, and their samples display greater morphological diversity. These conditions allow the network to more accurately recognize and segment such regions. Taken together, these factors lead to a significant drop in joint segmentation accuracy for horseshoe-shaped tunnels.

### 5.2. Mis-Segmentation of Power Track as Cable in Quasi-Rectangular Tunnels

In the segmentation results of this study, the network is able to accurately distinguish the major components within regular tunnel structures, achieving high segmentation accuracy across all categories. Nevertheless, in quasi-rectangular tunnel structures, a few instances of power tracks are misclassified as cables. This phenomenon mainly stems from both categories exhibit elongated and continuous geometric shapes in subway tunnel point clouds. In the absence of stable RGB information, relying solely on spatial structure and intensity features is insufficient to achieve robust discrimination. Furthermore, their practical layout is often adjacent or even intersecting, which further increases the likelihood of feature confusion. Overall, the current network has already demonstrated strong robustness and good generalization capability in complex tunnel environments. Future work will further broaden the diversity of data sources and optimize feature representations for geometrically similar categories, thereby enhancing the network’s performance in fine-grained structural discrimination.

### 5.3. Analysis of Model Complexity and Engineering Practicality

Beyond segmentation accuracy, the feasibility of point cloud semantic segmentation methods in real-world engineering applications also depends on their computational complexity and resource consumption. Therefore, this study further discusses the engineering practicality of the proposed MFPNet in terms of model parameter size, inference time, and GPU memory usage. Due to the introduction of the Multi-Scale KPConv module and related feature enhancement structures, MFPNet exhibits an increase in both model size and computational complexity compared with single-scale point cloud networks. The total number of parameters is approximately $2.0\times 10^{7}$ (around 20 M). This parameter increase mainly originates from the parallel multi-scale convolution branches and a small number of feature modulation structures, representing a direct consequence of multi-scale feature modeling. Although this design introduces more noticeable parameter and computational overhead than single-scale architectures, the increase in complexity has clear structural origins and maintains a reasonable engineering trade-off with the achieved performance gains. Regarding inference efficiency, owing to the irregular structure of point cloud data and the dynamic variation in the number of points per batch, the inference time of the model may fluctuate with input scale. Meanwhile, the additional computational overhead introduced by the multi-scale branches does not lead to unstable inference behavior, and the overall inference process remains controllable under a single-GPU setting. In terms of memory consumption, the GPU memory usage of MFPNet is mainly determined by the multi-scale neighborhood search process and the storage of intermediate feature maps. Under the experimental configuration adopted in this study, the model can be trained and inferred smoothly on a single GPU without introducing additional memory bottlenecks. Overall, although the multi-scale architecture increases computational and storage costs to some extent, it significantly enhances feature representation capability and segmentation performance while maintaining good computational efficiency and resource controllability, demonstrating the practical applicability of MFPNet in complex tunnel engineering scenarios.

## 6. Conclusions

The MFPNet proposed in this study is a deep learning network aimed at multi-class fine-grained semantic segmentation of tunnel structures. It addresses common challenges in underground environments, including large variations in structural scale, the tendency of small objects to be overlooked, and uneven point cloud density, by constructing an efficient feature extraction architecture that integrates multi-scale perception, attention enhancement, and semantic feedback. With the introduction of the multi-scale kernel point convolution module, MFPNet is capable of simultaneously capturing geometric features of both large-scale structures and small objects within point clouds under different receptive fields. The lightweight FMEM module dynamically adjusts responses in local key regions, thereby enhancing the recognition of fine structures such as cracks and bolts. In addition, the EFFM feedback module fuses deep and shallow semantic information, alleviating the semantic gap while maintaining computational efficiency, and significantly improves boundary recognition accuracy and semantic consistency.

Experimental results on the STSD tunnel point cloud dataset demonstrate that MFPNet significantly outperforms existing mainstream methods across multiple evaluation metrics, particularly exhibiting stronger robustness and fine-grained discriminative capability in the recognition of small target classes and weak boundary regions. Although the proposed multi-scale feature modeling and semantic enhancement strategies show a certain degree of potential for broader applicability at the methodological level, their effectiveness in more complex or substantially different point cloud scenarios still requires further validation through cross-dataset experiments. Overall, this study provides a technically viable solution with engineering reference value for the fine-grained semantic understanding and intelligent maintenance of tunnel and underground space infrastructures. It is worth noting that, despite achieving high accuracy in structurally regular subway tunnels with smooth inner linings, several limitations remain: First, in horseshoe-shaped tunnels where the transitions between joints and tunnel walls are smooth and geometric distinctions are less apparent, multi-modal data fusion strategies could be introduced to compensate for the shortcomings of single point cloud data in recognizing weak-feature categories. Second, the current work should be extended to cover a broader range of regular tunnel types, such as urban utility corridors and hydraulic tunnels, in order to improve the applicability and universality of the method. Third, future research may further expand its application scenarios by focusing on irregular environments such as underground mining roadways with rough surfaces and complex morphologies, thereby exploring the adaptability and robustness of the method under complex geological conditions.

## Figures and Tables

**Table 1:** Testing resulting.

Sets	Background	Rails Tracks	Power Track	Walkway	Pipe	Cable	Attachment	Big Bolt	Small Bolt	Signal Line	Joint
Precision (%)	98.5	95.4	99.3	97.9	98.9	93.6	90.9	93.2	83.6	90.9	61.3
Recall (%)	96.6	97.0	99.6	97.9	99.7	95.6	89.0	94.2	92.8	98.1	82.6
F1 Score (%)	97.5	96.2	99.4	97.9	99.3	94.6	89.9	93.7	87.9	94.4	70.4
IoU (%)	95.2	92.7	98.9	95.9	98.6	89.7	81.7	88.2	78.5	89.3	54.3

**Table 2:** Comparison results of different networks.

Class	PointNet++	DGCNN	SCF-Net	RandLA-Net	BAAF-Net	Point-Transformer	KPConv	MFP-Net
Background	72.0	74.0	82.3	80.3	80.5	81.0	93.6	95.2
Rails tracks	60.0	66.0	88.5	87.3	87.2	90.0	91.0	92.7
Power track	59.0	65.0	93.6	92.5	92.8	92.5	96.6	98.9
Walkway	68.5	73.0	84.6	84.5	83.0	85.0	94.3	95.9
Pipe	68.0	72.0	87.5	86.9	87.0	88.0	97.4	98.6
Cable	55.0	58.0	82.2	80.2	80.8	83.0	85.4	89.7
Attachment	36.7	48.0	62.0	53.9	57.1	69.0	76.6	81.7
Big Bolt	52.2	60.0	76.3	79.5	79.5	81.0	84.4	88.2
Small bolt	35.9	52.0	59.7	57.9	57.9	70.0	68.8	78.5
Signal line	62.0	70.0	77.7	75.0	76.3	78.5	84.0	89.3
Joint	26.5	32.0	37.6	34.7	34.6	38.0	34.3	54.3
mIoU(%)	54.5	61.8	75.6	73.9	74.2	77.8	82.4	87.5

**Table 3:** The results of the ablation study.

Module	Model						
	1	2	3	4	5	6	7
Multi-Scale KPConv	**✓**	**✓**	**✓**	**✗**	**✗**	**✗**	**✓**
EFFM	**✗**	**✓**	**✗**	**✓**	**✓**	**✗**	**✓**
FMEM	**✗**	**✗**	**✓**	**✗**	**✓**	**✓**	**✓**
mIoU (%)	84.7	85.2	85.5	84.4	86.0	83.0	87.5
Accuracy (%)	93.7	94.1	84.8	93.5	95.4	93.0	96.3

## Data Availability

The original data presented in the study are openly available in “https://github.com/lichking2017/STSD (accessed on 23 January 2025)”.
